# Deep Learning Models Used in the Diagnostic Workup of Keratoconus: A Systematic Review and Exploratory Meta-Analysis

**DOI:** 10.1097/ICO.0000000000003467

**Published:** 2024-02-01

**Authors:** Nicolas S. Bodmer, Dylan G. Christensen, Lucas M. Bachmann, Livia Faes, Frantisek Sanak, Katja Iselin, Claude Kaufmann, Michael A. Thiel, Philipp B. Baenninger

**Affiliations:** * Medical Faculty, University of Zurich, Zurich, Switzerland;; † Medignition Inc. Research Consultants Zurich, Zurich, Switzerland;; ‡ University of Toronto, Institute of Health Policy, Management and Evaluation (IHPME), Toronto, ON, Canada;; ¶ Department of Ophthalmology, Cantonal Hospital of Lucerne, Lucerne, Switzerland; and; § NIHR Biomedical Research Center at Moorfields Eye Hospital NHS Foundation Trust and UCL Institute of Ophthalmology, London, United Kingdom.

**Keywords:** artificial intelligence, deep learning, keratoconus, sensitivity and specificity, systematic review

## Abstract

**Purpose:**

The prevalence of keratoconus in the general population is reported to be up to 1 of 84. Over the past 2 decades, diagnosis and management evolved rapidly, but keratoconus screening in clinical practice is still challenging and asks for improving the accuracy of keratoconus detection. Deep learning (DL) offers considerable promise for improving the accuracy and speed of medical imaging interpretation. We establish an inventory of studies conducted with DL algorithms that have attempted to diagnose keratoconus.

**Methods:**

This systematic review was conducted according to the recommendations of the PRISMA statement. We searched (Pre-)MEDLINE, Embase, Science Citation Index, Conference Proceedings Citation Index, arXiv document server, and Google Scholar from inception to February 18, 2022. We included studies that evaluated the performance of DL algorithms in the diagnosis of keratoconus. The main outcome was diagnostic performance measured as sensitivity and specificity, and the methodological quality of the included studies was assessed using QUADAS-2.

**Results:**

Searches retrieved 4100 nonduplicate records, and we included 19 studies in the qualitative synthesis and 10 studies in the exploratory meta-analysis. The overall study quality was limited because of poor reporting of patient selection and the use of inadequate reference standards. We found a pooled sensitivity of 97.5% (95% confidence interval, 93.6%–99.0%) and a pooled specificity of 97.2% (95% confidence interval, 85.7%–99.5%) for topography images as input.

**Conclusions:**

Our systematic review found that the overall diagnostic performance of DL models to detect keratoconus was good, but the methodological quality of included studies was modest.

## Abstract 2

Supplemental Digital Content is Available in the Text.

Keratoconus is a progressive bilateral corneal ectasia resulting in corneal thinning, irregular astigmatism, and eventually visual impairment^1^ with a prevalence of up to 1:84.^2^ Keratoconus detection is important to halt potential disease progression. By performing corneal cross-linking, good vision can be preserved.^1^ In early disease, the condition may go undiagnosed unless assessment of posterior and anterior corneal surfaces is undertaken.^3^ The main diagnostic imaging techniques include corneal topography with Placido disc-based imaging systems (eg, Orbscan, Bausch & Lomb, Bridgewater, NJ), three-dimensional tomographic imaging such as Scheimpflug (eg, Pentacam, Oculus Optikgeräte GmbH, Wetzlar, Germany), and anterior segment optical coherence tomography (OCT).^4^ Keratoconus screening in clinical practice is still challenging and asks for improving the accuracy of keratoconus detection.^5^

Artificial intelligence (AI) has shown astonishing success with visual recognition tasks. Machine learning (ML) is an important branch in the field of AI and has the potential to automatically pinpoint, identify, and grade pathologic features in ocular diseases.^6^ Among the techniques comprising ML, one of the most promising is deep learning (DL).^5^ This mimics the operation of the human brain using multiple layers with an array of nodes/neurons that can generate automated predictions from input data.^6^ Although corneal tomography maps are not images of the eye per se, the color maps can be used to develop DL algorithms to detect keratoconus.^7^ Different AI approaches, such as feedforward neural networks, convolutional neural networks, support vector ML, and automated decision-tree classification algorithms, have been described.^4^ In this rapidly evolving field, meta-epidemiological summaries and appraisals of the underlying evidence would be useful for both researchers and clinicians. However, these overviews remain scarce and incomplete. Therefore, this systematic review inventoried and evaluated the currently available evidence reporting on the diagnostic performance of DL algorithms in diagnosing keratoconus.

## MATERIALS AND METHODS

This systematic review was conducted according to the Preferred Reporting Items for Systematic reviews and Meta-Analyses (PRISMA) statement recommendations.^8,9^ Methods of analysis and inclusion criteria were specified in advance and documented in registered protocol at the PROSPERO International prospective registry (CRD42020156004).

### Search Strategy

The complete search strategy is available on request. All empirically conducted studies from the following databases were searched: Ovid MEDLINE, Embase, Science Citation Index, Conference Proceedings Citation Index, and Google Scholar. In addition, we applied the search strategy to arXiv, a document server for preprints commonly used in the AI research community to publish study results. A parallel search of registered unpublished trials was conducted using clinical trials registers provided by the US National Library of Medicine (www.ClinicalTrials.gov), the WHO (www.who.int/ictrp/en/), the European Union (www.clinicaltrialsregister.eu), and the ISRCTN (www.isrctn.com) using the same restrictions and search criteria. Manual searches of bibliographies, citations, and “related articles” (PubMed function) of included studies were undertaken to identify any additional relevant articles that the searches might have missed. Date inclusions were from inception until February 18, 2022.

### Selection of Studies

Eligibility was assessed by 2 reviewers in pairs who screened titles and abstracts of the search results independently, with nonconsensus being resolved by a third reviewer. We included peer-reviewed studies that developed and evaluated a DL model for the diagnosis of keratoconus as an outcome in the field of imaging diagnostics using topography, tomography, or anterior segment OCT. We excluded studies that were not performed in the field of keratoconus, were performed in animals or with nonhuman samples, used only simulated data, or presented duplicate data. In addition, we excluded studies published in languages other than English, German, French, and Spanish. We did not impose any restrictions on publication status or study quality to capture gray literature and present all available evidence.

### Data Extraction and Management

Two reviewers (N.S.B. and P.B.B.) extracted data independently using a predefined data extraction sheet, cross-checked the data, and resolved disagreements by discussion or referral to a third senior reviewer (L.M.B.).

### Assessment of Methodological Quality

Two reviewers (N.S.B. and P.B.B.) independently assessed each report using the items proposed following recommendations by the quality assessment tool for diagnostic accuracy studies 2 (QUADAS-2) report.^10^ We adapted the QUADAS-2 report as certain categories (index test masked or threshold prespecified) are not applicable to our study types. This was cross-checked by a senior epidemiologist (L.M.B.). The reference standard (classification of absence or presence of disease) was considered appropriate when each image was classified by 2 independent, board-certified corneal specialists.

### Data Analysis and Synthesis

We summarized means and ranges of continuous variables and percentages of dichotomous variables. Where possible, we extracted binary diagnostic accuracy data and constructed contingency tables at the reported thresholds. Contingency tables consisted of true-positive, false-positive (FP), true-negative (TN), and false-negative results. Owing to methodological reasons, the minimum requirement to be included in the meta-analysis was at least 4 studies providing a 2-by-2 table. If multiple models were reported, we selected the main model, or if no primary model was identifiable, we selected the first model reported. We estimated and plotted summary receiver operating characteristics (ROC) curves using a unified model for meta-analysis of diagnostic test accuracy studies.^11^ We also indicated on the ROC figure the 95% confidence and prediction regions, providing estimates of average sensitivity and specificity across included studies. Analyses were performed using the Stata 16.1 statistics software package (StataCorp. 2019; StataCorp LLC, College Station, TX).

## RESULTS

### Study Selection

**FIGURE 1.**
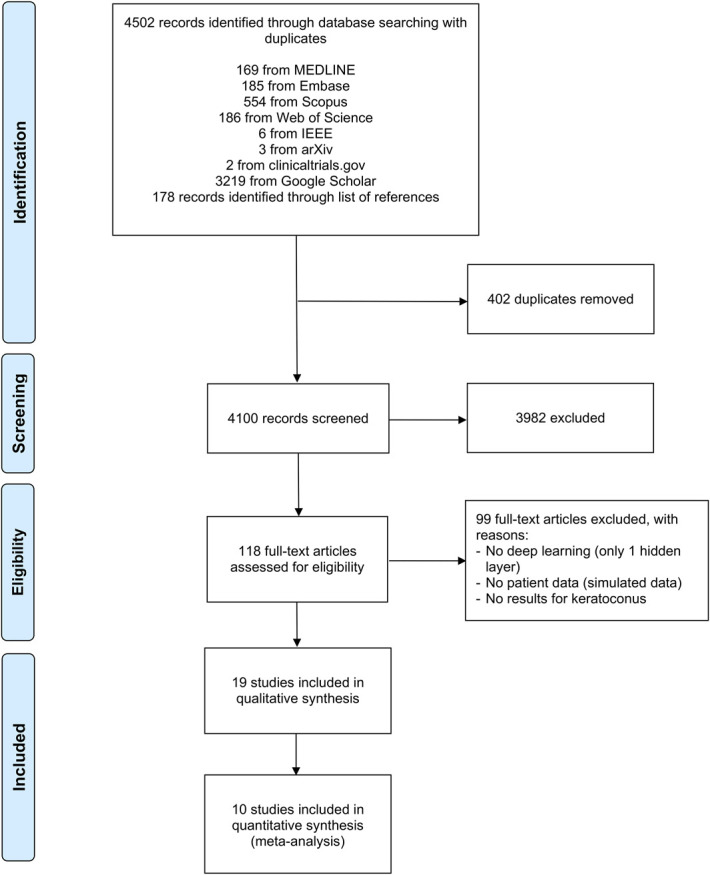
Study selection process according to the PRISMA statement.

Electronic searches retrieved 4324 studies, and additional 178 studies were identified from reference lists of review articles. After removing 402 duplicates, 4100 studies were screened for inclusion based on title and abstract. After excluding 3982 studies that did not fulfill the inclusion criteria, 118 studies were finally retrieved and assessed in full text. Of these, 19 studies met our inclusion criteria. Twelve studies allowed to extract contingency tables, and 10 were included in the quantitative analyses. The study selection process is outlined in Figure 1.

### Study Characteristics

We included 19 studies in the systematic review that contained 96,549 images or scans from over 10,500 patients.^5,7,12–28^ Five studies assessed the diagnostic performance of DL algorithms using anterior segment OCT images,^5,17–19,26^ 12 studies using corneal topography images (8 Scheimpflug and 4 Placido disk based),^7,12–16,20–24,28^ and 2 studies using smartphone images (topography^25^ and lateral segment images^27^). Keratoconus grading was reported in 11 studies, 4 of which used the Amsler-Krumeich classification.^5,16,22,23^ Only 7 of 19 studies provided a description of the study population including corneal characteristics. All salient study characteristics are summarized in Tables 1 and 2.

**TABLE 1.** Summary of Study Design, Imaging Modality, and Inclusion and Exclusion Criteria

Author, yr	Validation	Study Design	Imaging Modality	Keratoconus Grading	Inclusion/Exclusion Criteria	Reference Test	Transfer Learning	Training Set	Validation Set	Test Set	Low-Quality Images Excluded	Patient Baseline Characteristics Reported	Number of Patients (Eyes)
Abdelmotaal et al^13^	Split sample	n.r.	Scheimpflug corneal topography (Pentacam, Oculus Optikgeräte GmbH, Wetzlar, Germany)	Healthy, subclinical KC, KC	Imaging was performed between July 2014 and March 2019. Refractive surgery candidates (refractive error of <8.0 D sphere, with <3.0 D of astigmatism, without clinical, topographic, or tomographic signs of KC), patients with unilateral or bilateral KC (clinical diagnosis of KC, irregular cornea determined by distorted keratometry mires or distortion of retinoscopic red reflex, typical topographic findings), patient with subclinical KC (subtle corneal topographic changes in the aforementioned KC abnormalities in the absence of slitlamp or visual acuity changes typical of KC)	Two corneal specialists independently classified the anonymized images. A third party resolved conflicts (differences in labeling)	No	Training + validation (split sample: 0.3 validation set) Total: 2574 images/eyes • Healthy: 887 • Subclinical KC: 857 • KC: 830		Total: 644 • Healthy: 221 • Subclinical KC: 215 • KC: 208	n.r.	Age, corneal characteristics	Total: 1669 (3218)
Abdelmotaal et al^12^	Split sample	n.r.	Scheimpflug corneal topography (Pentacam)	Healthy, early KC, KC	Refractive surgery candidates, and patients with unilateral or bilateral keratoconus, obtained between July 2014 and March 2019. Facilitated by anonymized clinical examination charts, the anonymized images were classified into keratoconus (K), early keratoconus (E), and normal (N) groups, using the following criteria: keratoconus group (K), those with a clinical diagnosis of keratoconus such as (a) the presence of a central protrusion of the cornea with Fleischer ring, Vogt striae, or both by slit-lamp examination or (b) an irregular cornea determined by distorted keratometry mires and distortion of retinoscopic red reflex or both. In addition, the K group included the following topographic findings as summarized by Pi’nero and colleagues: focal steepening located in a zone of protrusion surrounded by concentrically decreasing power zones, focal areas with dioptric (D) values >47.0 D, IS asymmetry measured to be >1.4 D, or angling of the hemimeridians in an asymmetric or broken bowtie pattern with skewing of the SRAX. Early keratoconus group (E) was defined as subtle corneal tomographic changes as the aforementioned keratoconus abnormalities in the absence of slitlamp or visual acuity changes typical of keratoconus. Normal group (N) comprised refractive surgery candidates and subjects applying for a contact lens fitting with a refractive error of <8.0 D sphere with <3.0 D of astigmatism and without clinical, topographic, or tomographic signs of keratoconus or early keratoconus	Two corneal specialists independently classified the anonymized images. A third party resolved conflicts (differences in labeling)	Yes	Training + validation (Split sample: 0.3 validation set) Total: 1688 images/eyes • Healthy: 860 • Early KC: 554 • KC: 274		Total: 90 • Healthy: 30 • Early KC: 30 • KC: 30	n.r.	Age, corneal characteristics	Total: 923 (1778)
Al-Timemy et al^15^	Cross-validation (5-fold) + separate test set	n.r.	Scheimpflug corneal topography (Pentacam)	Healthy, suspected KC, KC	Eyes were labeled as suspected KC if the corneal topography included atypical, localized steepening or an asymmetrical bowtie pattern. Eyes were labeled as suspected KC if the keratometric curvature was >47.0 diopters, the oblique cylinder was more than 1.5 D, or the central corneal thickness was below 500 micrometer. Eyes were labeled as suspected KC when BAD-D was between 1.6 and 3.0 The independent validation subset included 150 eyes of 85 patients collected from Hospital de Olhos–CRO, a private hospital located in São Paulo, Brazil	Three corneal-trained specialists performed the eye image classification. Disagreements were dealt favoring 2 versus 1 vote	Yes	Training + validation (cross-validation 5-fold). Total: 542 eyes • Healthy: 204 • Suspected KC: 123 • KC: 215		Total: 150 eyes • Healthy: 50 • Suspected KC: 50 • KC: 50	n.r.	Age, corneal characteristics	Total: 280 (542) • Healthy: 104 (204) • Suspected KC: 63 (123) • KC: 113 (215) + test set 85 (150)
Al-Timemy et al^14^	Split sample	n.r.	Scheimpflug corneal topography (Pentacam)	Healthy (included forme fruste), KC (included ectasia + KC)	The data set used in this study is based on patients preassessed with KC and normal participants referred to Al-Amal ophthalmic center in Baghdad, Iraq. Normal and subclinical cases, where the readings were similar to normal and the disease is stopped and did not progress. Subclinical cases can be regarded as a cornea with no abnormal findings which is confirmed by both corneal topography and slit-lamp examination. The decision of KC was supported by subjective and slit-lamp examination. Any patient with other ocular diseases was excluded from the study	The classification of the 2 groups was screened by an ophthalmologist and ophthalmology supervisor.	Yes	Total: 355 eyes • Healthy: n.r. • KC: n.r. (partially augmented data set)	Total: 63 eyes • Healthy: n.r. • KC: n.r. (partially augmented data set)	Total: 116 eyes • Healthy: 56 • KC: 60	n.r.	Age	Total: n.r. (444) (90 KC in a training/validation set were augmented)
Chen et al^16^	Split sample + additional test set	Case–control study	Scheimpflug corneal topography (Pentacam)	Amsler-Krumeich classification: Healthy, Stage 1, Stage 2, Stage 3, Stage 4	Tomographic images of keratoconic and healthy eyes provided by 3 centers: The Royal Liverpool University Hospital (UK); Sedaghat Eye Clinic, Mashhad (Iran), and The New Zealand National Eye Center (New Zealand). Patients with all stages of keratoconus were included. Images were obtained between January 2013 and January 2020. For the control group, subjects with a Belin/Ambrósio Enhanced Ectasia total deviation index (BAD-D) of < 1.6 SD from normative values (indicating the absence of ectasia) were included	n.r.	Yes	Training + validation (Split sample: 0.2 validation set) Total: 1115 eyes • Healthy: 82 • Stage 1: 159 • Stage 2: 211 • Stage 3: 115 • Stage 4: 548		Split sample test set: 279 eyes • Healthy: 20 • Stage 1: 40 • Stage 2: 53 • Stage 3: 29 • Stage 4: 137 Second (external) test set: 532 eyes • Healthy: 32 • Stage 1: 83 • Stage 2: 161 • Stage 3: 64 • Stage 4: 192	Yes.	n.r.	Total: 1836 (1926)
Elsawy et al^18^	Split sample + additional test set	Case–control study	Anterior segment OCT (Envisu R2210, Bioptigen, Buffalo Grove, IL)	Healthy, KC, Fuchs endothelial dystrophy, Dry eye	The patients were recruited at BPEI and signed a written informed consent form approved by the University of Miami Institutional Review Board. Either dry eye, Fuchs endothelial dystrophy, healthy patient or patient with keratoconus. Exclusion: The OCT images with bad quality were excluded	The OCT images were assessed by corneal specialists	Yes	Total: 21,388 images • Healthy: 5038 • KC 5750 • Fuchs endothelial dystrophy: 5190 • Dry eye: 5410	Total: 2674 images • Healthy: 630 • KC 719 • Fuchs endothelial dystrophy: 648 • Dry eye: 677	Total: 2674 images • Healthy: 630 • KC 719 • Fuchs endothelial dystrophy: 649 • Dry eye: 676 + additional test set: 40 images of 40 eyes of 20 patients	Yes	n.r.	Total: 269 (413) • Healthy: 77 (121) • KC 109 (162) • Fuchs endothelial dystrophy: 51 (80) • Dry eye: 32 (50) + additional test set: 20 (40)
Elsawy et al^19^	Split sample + separate test set	Cohort study	Anterior segment OCT (Envisu R2210)	Healthy KC	Prospectively recruited from June 2016 to September 2019 at the Bascom Palmer Eye Institute. Healthy patient was defined as having no *ICD*-10 corneal diagnoses, KC was defined as having an *ICD*-10 diagnosis of KC (H18.603). Separate test set: independent clinical study patient set of different consecutive patients Exclusion: Patients with other *ICD*-10 corneal pathologies were excluded from all groups.	All participants underwent an ophthalmologic examination by 1 of 6 fellowship-trained corneal specialists	Yes	Total: 17,934 images • Healthy: 8855 • KC: 9079 Training set: 14,347 • Healthy: n.r. • KC: n.r. Validation set: 3587 • Healthy: n.r. • KC: n.r.		Separate test set: Total: 15,300 images • Healthy: 7560 • KC: 7740	Yes	Age, sex	Total: n.r. (n.r.) + separate test set: 44 (85)
Elsawy et al^17^	Cross-validation	n.r.	Anterior segment OCT (Envisu R2210)	Healthy, KC	The patients were recruited at the BPEI	OCT scans were assessed by corneal specialists	Yes	5-fold cross-validation, where 4 folds were used for training, and 1-fold was used for validation. Total: 437 scans • Healthy: 131 • KC: 306		n.a.	Yes	n.r.	Total: 228 (373) • Healthy: 67 (112) • KC: 161 (261)
Feng et al^20^	Split sample and cross-validation	n.r.	Scheimpflug corneal topography (Pentacam)	Healthy, subclinical KC, KC	Patients with KC and subclinical KC, as well as healthy patients, were included. Only 1 eye of each patient was included	n.r.	No	Split sample 0.2 test set. Cross-validation for training set. Total: 854 eyes • Healthy: 335 • Subclinical KC: 172 • KC: 347			n.r.	Age, sex, corneal characteristics	Total: 854 (854)
Gandhi et al^21^	Cross-validation	n.r.	Placido disk-based corneal topography (ATLAS 9000, Carl Zeiss Meditec AG, Jena, Germany)	Healthy, KC (moderate + severe)	n.r.	n.r.	Yes (VGG16)	10-fold cross-validation Total: 1104 eyes • Healthy: 300 • KC: 804		n.a.	n.r.	n.r.	Total: 552 (1104)
Hallett et al^22^	Split sample	Cohort study	Scheimpflug corneal topography (Pentacam) parameters (not images) + 20 clinical parameters	Amsler-Krumeich classification: Healthy, Stage 1, Stage 2, Stage 3, Stage 4	This is a retrospective single-center study. Patient data from 124 KC patients were collected from Vision Eye Institute Chatswood between 2014 and 2017	n.r.	n.r.	Total: 170 samples Total: n.r. • Healthy: n.r. • Stage 1: n.r. • Stage 2: n.r. • Stage 3: n.r. • Stage 4: n.r.	Total: 43 samples Total: n.r. • Healthy: n.r. • Stage 1: n.r. • Stage 2: n.r. • Stage 3: n.r. • Stage 4: n.r.	Total: 24 samples • Healthy: n.r. • Stage 1: n.r. • Stage 2: n.r. • Stage 3: n.r. • Stage 4: n.r.	n.r.	Sex	Total: 124 (n.r.)
Kamiya et al^5^	Cross-validation	Case–control study	Anterior segment OCT (SS-1000, Tomey, Aichi, Japan)	Amsler-Krumeich classification: Healthy, Stage 1, Stage 2, Stage 3, Stage 4	Patients were recruited between March 2013 and April 2018 at Miyata Eye Hospital. Keratoconus was diagnosed by corneal specialists with evident topography findings and at least 1 keratoconus sign (stromal thinning, conical protrusion of the cornea at the apex, Fleischer ring, Vogt striae, or anterior stromal scar) on slit-lamp examination. Healthy eyes had normal topography and ocular findings applying for a contact lens fitting or refractive surgery consultation. The control subjects had a refractive error (spherical equivalent) of < 6 diopters (D) and/or astigmatism of <3 D. The patients who wore rigid and soft contact lenses were asked to stop using them for 3 and 2 wks, respectively, before this assessment. Exclusion: Other corneal diseases such as pellucid marginal degeneration and eyes with a history of trauma or corneal surgery such as corneal cross-linking for progressive keratoconus were excluded from the study	Diagnosis made by corneal specialists	Yes	5-fold cross-validation Total: 543 eyes • Healthy: 239 • Stage 1: 108 • Stage 2: 75• Stage 3: 42 • Stage 4: 79		n.a.	n.r.	n.r.	Total: n.r. (543)
Kamiya et al^23^	Cross-validation	Case–control study	Placido disk-based corneal topography (TMS-4, Tomey, Aichi, Japan)	Amsler-Krumeich classification: Healthy, Stage 1, Stage 2, Stage 3, Stage 4	Good quality images of corneal topography measured with a Placido disk corneal topograph were included in this study. Multiple corneal specialists diagnosed keratoconus with distinctive features (e.g., corneal color-coded map with an asymmetric bowtie pattern with or without skewed axes), and at least 1 keratoconus sign (e.g., stromal thinning, conical bulging, Fleischer ring, Vogt striae, or apical scar). We used the Amsler-Krumeich classification to evaluate the grade of the keratoconus. As a control group we examined subjects with normal ocular findings applying for a contact lens fitting or for a refractive surgery consultation, who had a refractive error of <6 diopters (D) as well as astigmatism of <3 D	Multiple corneal specialists diagnosed keratoconus	Yes	7-fold cross-validation (where 5 folds were used for training, 1-fold was used for validation, and 1-fold was used for testing after the training finished) Total: 349 eyes • Healthy: 170 • Stage 1: 54 • Stage 2: 52 • Stage 3: 23 • Stage 4: 50			Yes	Age, corneal characteristics	Total: n.r. (349) • Healthy: n.r. (170) • KC: n.r. (179)
Kuo et al^24^	Split sample	Case–control study	Placido disk-based corneal topography (TMS-4)	Healthy, subclinical KC, KC	Patients were recruited from 2007 to 2020. Criteria for diagnosing manifested keratoconus were defined per clinical findings and topographic criteria previously described. Clinical signs of keratoconus were the existence of central protrusion of the cornea, Fleischer ring, Vogt striae, and focal corneal thinning on slit-lamp examination. Topographic criteria were central K value >47 diopter, I-S value >1.4 diopter, keratoconus percentage index (KISA%) >100%, and asymmetric bowtie presentation. The main criteria of subclinical keratoconus were based on the topographic pattern. Asymmetric bowtie with skewed radial axes (AB/SRAX), asymmetry bowtie with inferior steep (AB/IS), and symmetric bowtie with skewed radial axes (SB/SRAX) presented in the topography and no slit-lamp keratoconus findings were collected. The best spectacle–corrected vision of patients with subclinical keratoconus was not affected. The control group comprised candidates for refractive surgery without any manifestations earlier, with regular astigmatism. Patients with previous eye surgery, ocular trauma, contact lens discontinuation <3 wk, and younger than 20 years were excluded	Four corneal specialists involved with the classification	Yes	Total: 254 images • Healthy: 120 • KC: 134	Total: 72 images • Healthy: 36 • KC: 36 +28 images of separate subclinical KC dataset		n.r.	Age, sex, corneal characteristics	Total: 206 (n.r.) • Healthy: 84 (n.r.) • Subclinical KC: 28 (n.r.) • KC: 94 (n.r.)
Lucena et al^25^	Split sample	n.r.	Topography smartphone image	Spherical pattern, regular symmetrical astigmatism, regular asymmetrical astigmatism, KC	n.r.	Classification performed by specialist	No	Total: 960 images • Spherical pattern: 240 • Regular symmetrical astigmatism: 240 • Regular asymmetrical astigmatism: 240 • KC: 240	Total: 212 images • Spherical pattern: 35 • Regular symmetrical astigmatism: 62 • Regular asymmetrical astigmatism: 55 • KC: 60		n.r.	n.r.	n.r. (n.r.)
Mahmoud et al^26^	Cross-validation	n.r.	Anterior segment OCT (SS-1000)	Healthy, mild KC, moderate KC, severe KC	n.r.	Extraction of medical diagnoses conducted by a physician	No	10-fold cross-validation Total: 268 images • Healthy: 100 • Mild KC: 58 • Moderate KC: 70 • Severe KC: 40			n.r.	n.r.	n.r. (n.r.)
Xie et al^28^	Split sample	Cohort study + case-control study for test set	Scheimpflug corneal topography (Pentacam)	Healthy, suspected irregular cornea, early-stage KC, KC, myopic postoperative cornea	The sample population was patients throughout China who wanted to undergo refractive surgery, had a primary diagnosis of KC, and had stable postoperative refractive states	Each image was independently labeled by 3 senior ophthalmologists with at least 5 y of practical experience in the refractive surgery center. When the labels differed, the 1 chosen by 2 of the 3 experts was selected	Yes	Total: 5130 Images • Healthy: n.r. • Suspected irregular cornea: n.r. • Early-stage KC: n.r. • KC: n.r. • Myopic postoperative cornea: n.r.	Total: 1335 images • Healthy: n.r. • Suspected irregular cornea: n.r. • Early-stage KC: n.r. • KC: n.r. • Myopic postoperative cornea: n.r.	Separate test set Total: 100 images • Healthy: 20 • Suspected irregular cornea: 20 • Early-stage KC: 20. • KC: 20 • Myopic postoperative cornea: 20	n.r.	n.r.	Total: 1385 (2802) + separate test set: 94 (n.r.)
Zaki et al^27^	Split sample	n.r.	LSPIs captured with a smartphone	Healthy, KC	The data set was collected at the ophthalmology department in HKL, Malaysia, with an appointed optometrist to validate the KC and healthy eyes using OCULUS Easygraph Topographer	The optometrist validated each subject's eye image based on the topographic map	Yes	Total: 2400 images • Healthy: 1200 • KC: 1200	Total: 800 images • Healthy: 400 • KC: 400	Total: 800 images • Healthy: 400 • KC: 400	n.r.	n.r.	Total: 250 (n.r.) • Healthy: 125 (n.r.) • KC: 125 (n.r.)
Zéboulon et al^7^	Split sample and cross-validation	Case–control study	Placido disk-based corneal topography (Orbscan, Bausch & Lomb, Bridgewater, NJ)	Healthy, KC	The preselection criteria for keratoconus were an anterior curvature map showing one of the classic keratoconus patterns described by Rabinowitz et al associated with corneal thinning. RS examinations' criteria were an oblate anterior surface (flat in its center), a prolate posterior surface (steep in its center), a central corneal thinning and lower central curvature values compared with the periphery (cases of myopic laser surgery). Finally, normal examinations were preselected if no corneal condition could be detected. If the case seemed to match 1 of the 3 desired diagnoses, the patient's electronic file was retrieved to confirm the diagnosis, and the examination was excluded or included in the study data set. This process was iterated until the target number of 3000 examinations evenly distributed was reached. Exclusion: Bad quality examinations with many artefacts or too many missing values were not included	Topography images were preselected by a resident and reviewed by a corneal tomography expert with at least 5 y of experience	n.r.	10-fold cross-validation Total: 1800 images/eyes • Healthy: 900 • KC: 900		Total 200 • Healthy: 100 • KC: 100	Yes	Age, sex, corneal characteristics	Total: 2000 (2000) • Healthy: 1000 (1000) • KC: 1000 (1000)

BPEI, Bascom Palmer eye Institute; HKL, Hospital Kuala Lumpur; KC, Keratoconus; LSPI, lateral segment photographed images; n.r., not reported; SRAX, steepest radial axis.

**TABLE 2.** Performance of DL Algorithms Under Investigation

Author, yr	Data Set Used for Performance Assessment	Sample Description	Image Modality	Deep Learning Model Architecture	Number of Models Tested	Sensitivity	Specificity	AUC (95% CI)	Accuracy	Meta- Analysis Incl.
Abdelmotaal et al^13^	Split sample: test set (20% of original sample)	Total: 644 eyes • Healthy: 221 • Subclinical KC: 215 • KC: 208	Scheimpflug corneal topography images	The CNN consists of 2 convolutional layers. A flatten layer is then used to allow feeding of the fully connected layer. This is followed by 4 similar stacks of fully connected (Dense 1, 2, 3, and 4) and dropout layers. Each fully connected layer contains 128 fully connected neurons and uses the ReLU activation function, followed by dropout regularization with 20% probability (0.2). The final (classifying) layer of the architecture is a fully connected layer (Dense 5) with SoftMax activation that contains 3 output neurons, resulting in the probability of classifying each of the image groups	5				0.989	
Abdelmotaal et al^12^	Test set (90 randomly selected images of original sample)	Total: 90 eyes • Healthy: 30 • Early KC: 30 • KC: 30	Scheimpflug corneal topography images	The pretrained VGG16 deep convolutional neural network with ImageNet weights was used and customized for image classification. After modifying the input tensor shape of the top dense layer, thereby forcing the model to accept the shape 512 × 512 of the input images, the last classifying layers of the model were truncated and replaced by a flattened layer followed by 2 fully connected layers (64 nodes- Denses 1 and 2) separated by a dropout layer and followed by a final fully connected (3 nodes- Dense 3) layer with SoftMax activation adapted to output the 3 image classes. To enhance performance, pix2pix conditional generative adversarial network (pix2pix cGAN) was used to create plausible synthesized Scheimpflug camera color-coded corneal tomography images based on a modest-sized original data set for training the model. Six different data sets were assessed	Six augmented training data sets were tested (same model). Balanced original data set extracted				0.973	
Al-Timemy et al^15^	Test set (100 eyes. 7 maps per eye included)	Total: 100 eyes • Healthy: 50 • KC: 50	Scheimpflug corneal topography images	Hybrid model integrating information from 7 models based on EfficientNet-b0, a deep learning architecture, pretrained on ImageNet, to identify KC-induced signs in each corneal map separately. EfficientNet-b0 is a DL architecture with 290 layers. A support vector machine classifier was used for final classification	1			0.990	0.920	
Al-Timemy et al^14^	Split sample: test set (22% of original sample)	Total: 116 eyes • Healthy: 56 • KC: 60	Scheimpflug corneal topography images	Four pretrained networks, SqueezeNet (SqN), AlexNet (AN), ShuffleNet (SfN), and MobileNet-v2 (MN), and fine-tune them on a data set of KC and normal cases, each including 4 topographic maps. We also consider a PI classifier. Then, the EDTL method combines the output probabilities of each of the 5 classifiers to obtain a decision based on the fusion of probabilities. A logistic regression classifier with a Stochastic Gradient Descent optimizer (LRSGD-PI) was used to classify the PI	Five (4 deep learning models + logistic regression classifier)				0.892 (SqN)^†^ 0.899 (AN) 0.860 (SfN) 0.864 (MN)	
Chen et al^16^	Additional test set (532 eyes)	Total: 532 eyes • Healthy: 32 • KC Stage 1–4: 500	Scheimpflug corneal topography images	Adapted VGG16 model, but to prevent overfitting, the number of connected weights of the top layer was reduced. Everything before the flattened layer was the same as the standard VGG16. After the flattened layer for the 2-class task and to fully connect the (FC) layer with 128 outputs with a rectified linear unit (ReLU), an FC layer with 64 outputs with ReLU and an FC layer with 2 outputs (the final output layer representing 2 classes) were used with a SoftMax activation function. After the flattened layer for the 5-class task, the order was an FC layer with 128 outputs, a ReLU dropout layer with probability 0.5, an FC layer with 64 outputs with ReLU dropout layer with probability 0.5, and an FC layer with 5 outputs (the final output layer representing 5 classes) were used with a SoftMax activation function	6 (only results for concentrated models are presented, as only this model was tested on external validation set)	0.996	0.625	0.974 (0.965–0.982)	0.974	X
Elsawy et al^18^	Additional test set (40 eyes)	Total: 40 eyes • Healthy: n.r. • KC: n.r. • Dry eye: n.r. • Fuchs endothelial dystrophy: n.r.	Anterior segment OCT	The authors used transfer learning and fine-tuning with pretrained deep learning classification networks, namely, AlexNet, VGG16, and VGG19 networks to create the disease classification networks	3				0.8500 (AlexNet) 0.9000 (VGG16) 0.9000 (VGG19)	
Elsawy et al^19^	Separate test set (85 eyes)	Total: 85 eyes • Healthy: 42 • KC: 43	Anterior segment OCT	A pretrained VGG19 network for the diagnosis of corneal diseases versus healthy controls was used. The VGG19 network consists of 14 convolutional layers for feature extraction, 5 MaxPooling layers for feature reduction (i.e., maximum pooling), 2 fully connected layers of size 4096, and a classification layer with a SoftMax activation function and 1000 categories	1	0.977	0.905	0.980 (0.950–1.000)	0.941	X
Elsawy et al^17^	Cross-validation	Total: 437 scans • Healthy: 131 • KC: 306	Anterior segment OCT	Adapted VGG16 network, using the convolutional layers of a pretrained VGG16 network as the encoder of our network. The multiresolution feature maps are reduced, depth-wise, using a point-wise convolutional layer with a linear activation function. A fully connected layer with 1024 neurons and a ReLU activation function is used to generate dense features of size 1024 × 1. Composite classification features are obtained by concatenating the multiresolution convolutional features and the dense features. Finally, a classification layer with 3 neurons representing the output classes and a SoftMax activation function was added	1	0.941	0.908		0.931	X
Feng et al^20^	Split sample: test set (20% of original sample)	Total: nr • Healthy: n.r. • Subclinical KC: n.r. • KC: n.r.	Scheimpflug corneal topography images	KerNet, containing 5 branches as the backbone with a multilevel fusion architecture. The 5 branches receive 5 matrices separately and capture effectively the features of different matrices by several cascaded residual blocks. The multilevel fusion architecture (i.e., low-level fusion and high-level fusion) moderately takes into account the correlation among 5 slices and fuses the extracted features for better prediction	1	0.937			0.947	
Gandhi et a^21^	Cross-validation	Total: 1104 eyes • Healthy: 300 • KC: 804	Placido disk-based corneal topography images	Artificial neural network (ANN)-tailored architecture uses an input layer 2 hidden layers with 64 and 16 neurons respectively along with an ‘ReLU’ activation function in each of the layers and an output layer with sparse categorical cross entropy function, to calculate the loss between predicted and actual labels. The input to the CNN (ConvNet) is color images of 534 × 534 × 3 pixels. This CNN model uses 3 × 3 kernel to execute convolution process. The CNN model consists of 10 hidden layers of which 4 layers use 32 filters and layer 5 uses 16 filters. In convolutional layers, padding and stride size are set as 1 for a training process that preserves useful information and decreases the dimension of data. Furthermore, feature data are dealt by flatten layer and subsequently, the most relevant and potential features are handed over to the fully connected output layer that is a single-dimensional vector ready for classifying data among keratoconus and healthy, cornea. The pretrained VGG16 uses 3 × 3 filter with stride value 1 and same padding and structure of max pooling layers with 2 × 2 filter and stride size 2. VGG16 uses 16 layers with weights and fully connected layer with a SoftMax function to produce output resulted in a very large network with approximately 138 million parameters. VGG16 uses 64 neurons in input layer and settles with 512 neurons in last hidden layer. All 3 models are evaluated with 3 different data set inputs: original color, and 2 adapted versions: edges and mask (color) and canny edges (binary)	Nine (results are extracted for original color data sets.)	0.960 (ANN) ^†^ 0.957 (CNN) 0.989 (VGG16)	0.913 (ANN) ^†^ 0.957 (CNN) 0.977 (VGG16)		0.94649 (ANN)^†^ 0.95742 (CNN) 0.98550 (VGG16)	X
Hallett et al^22^	Split sample: test set (10% of original sample)	Total: 24 samples • Healthy: n.r. • Stage 1: n.r. • Stage 2: n.r. • Stage 3: n.r. • Stage 4: n.r.	Scheimpflug corneal topography parameters (not images) + 20 clinical parameters	MLP with 3 hidden layers used for corneal data classification. The MLP model is a fully connected deep neural network with multiple layers for a semisupervised learning task. The network architecture is 29-128-256-4, with 29 the dimension of the input data (i.e., the number of the variables of corneal data) and 128; 256 and 4 the numbers of neurons in the hidden layers in turn.	1				0.739*	
Kamiya et al^5^	Cross-validation	Total: 543 eyes • Healthy: 239 • KC Stage 1–4: 304	Anterior segment OCT	Pretrained convolutional neural network ResNet-18 (open-source deep learning platform: PyTorch) from the ImageNet database was used. The authors separately trained 6 neural networks from each image of 6 color-coded maps (anterior elevation, anterior curvature, posterior elevation, posterior curvature, total refractive power, and pachymetry map) without a color-scaled bar. Each network classifies an image into 0–4. We integrated these 6 outputs by averaging them. For example, if these 6 classifiers' outputs are 2, 2, 2, 3, 4, and 3, their average is 2.67, resulting in an integrated result of 3. We applied a floor function for values such as 2.5, that is, it was interpreted as 2	1	0.984	1.000		0.991	X
Kamiya et al^23^	Cross-validation	Total: 349 eyes • Healthy: 170 • KC Stage 1–4: 179	Placido disk-based corneal topography images	Pretrained VGG16 network model using an open-source deep learning platform (PyTorch). This model has been pretrained by 3390 color-coded map images that were composed of anterior and posterior elevation, anterior and posterior curvature, total refractive power, and pachymetry maps obtained with an anterior segment OCT. Each network classifies an image into 0–4. The output value of neural network for an image is a real number, so that we aligned it to the nearest integer value to interpret. For example, if the output value is “2.67,” it is interpreted as “3” (classified as Grade 3)	1	0.944	0.988		0.966	X
Kuo et al^24^	Split sample: validation set	Total: 72 images • Healthy: 36 • KC: 36	Placido disk-based corneal topography images	Three well-known CNN models were adopted for transfer learning and analysis, namely, the VGG16 model, InceptionV3 model, and ResNet152 model. The VGG16 model was mainly stacked with a series of convolutional and pooling layers for image feature extraction and then connected to the fully connected layer for classification, which was viewed as the extension of the classic AlexNet model. The InceptionV3 model reorganized the common convolutional and pooling layers into the so-called Inception module, which comprised 3 convolutions and 1 pooling to widen the network layer to acquire more detailed image features and improve prediction accuracy. The ResNet152 model introduced the shortcut connection before and after convolutional layers to make a plain network into a residual network, which can build an ultra-deep network without problems of gradient vanishing or exploding to gain accuracy from the considerably increased depth	3	0.917 (VGG16)^†^ 0.917 (InceptionV3) 0.944 (ResNet152)	0.944 (VGG16)^†^ 0.944 (InceptionV3) 0.972 (ResNet152)	0.956 (VGG16)^†^ 0.987 (InceptionV3) 0.995 (ResNet152)	0.931 (VGG16)^†^ 0.931 (InceptionV3) 0.958 (ResNet152)	X
Lucena et al^25^	Split sample: validation set	Total: 157 • Spherical pattern: 35 • Regular symmetrical astigmatism: 62 • KC: 60	Corneal topography images captured by smartphone	TopEye model: no model architecture reported	1	0.950	0.948		0.949	
Mahmoud et al^21^	Cross-validation	Total: 268 • Healthy: 100 • KC: 168	Anterior segment OCT	The convolutional neural network consists of 4 convolutional, 4 max pooling, and 2 fully connected layers. The kernels sizes are: 3 × 3 × 3 in the convolutional layers and 2 × 2 × 2 in the pooling layers. The feature kernels are 96, 128, 256, and 512 in the convolutional layers, respectively. In the first convolutional layer, 3 × 3 × 3 96 filters are applied to 227 × 227 × 227 input images. The max pooling layer applies 2 × 2 × 2 filters to reduce the size of the preceding convolutional layer output. The reduced images are handled by the following convolutional layers after applying the second and third pooling layer. The model applies the rest of the layers until finally reaching the 2 fully connected layers with all neurons connected to neurons of the previous fully connected layer. A SoftMax classifier is used to classify the 3D corneal images. The output of the last fully connected layer is fed to the SoftMax classifiers as an input	1	0.952	0.960		0.955	X
Xie et al^28^	Separate test set (80 images)	Total: 80 images • Healthy + suspected irregular cornea: 40 • Early-stage KC + KC: 40	Scheimpflug corneal topography images	Pretrained InceptionResNetV2 architecture was used in a convolutional neural network on the TensorFlow platform. During the training process, only the weights for fully connected layers were updated in our training data set, and the other weights were pretrained on ImageNet and frozen	1	0.951	1.000		0.976	X
Zaki et al^27^	Split sample: test set (20% of original sample)	Total: 800 images • Healthy: 400 • KC: 400	Smartphone lateral segment photographed images	The pretrained VGGNeT-16 model already has rectified linear unit (ReLU) activation function and its optimizer; thus, it is not required to tune the activation function and its optimizer during the training and model generation. First, all LSPIs are loaded into the model and preprocessed. During the preprocessing step, the images are augmented to increase the number of training data sets artificially. Then, the pretrained model is called to load the pretrained weight. The first 5 bottom layers are frozen, while another 11 layers are unfreezed to create a new KC detection model	2	0.898	0.985		0.941	
Zéboulon et al^7^	Split sample: test set (10% of original sample)	Total: 200 images/eyes • Healthy: 100 • KC: 100	Placido disk-based corneal topography images	Convolutional neural network with an architecture inspired by the ResNet26 with fewer hidden layers and 4 input channels. The numeric data from 4 commonly used maps in clinical practice were selected for each examination: “Elevation against the anterior best fit sphere (BFS),” “elevation against the posterior BFS,” “axial anterior curvature,” and “pachymetry.” In an attempt to mimic the human diagnostic process, we specifically chose these 4 maps as they are the most commonly used routinely in this institution	1	1.000	1.000		1.000	X

A-K classification, Amsler-Krumeich classification; AUC, area under the curve; CNN, convolutional neural network; EDTL, Ensemble of Deep transfer Learning; KC, keratoconus; MLP, multilayer perceptron; n.r. = not reported; PI, Pentacam indices; RS, refractive surgery.

* No detailed results are available to calculate Healthy versus KC accuracy. Only accuracy for complete A-K classification is available.

† Selected model for exploratory meta-analysis.

### Methodological Characteristics

The overall quality assessed by adapted QUADAS-2^10^ was modest. Of the 19 included publications, only 3 were cohort studies and all were retrospective. Six studies used a case–control design, and in 10 studies, it was unclear how patients were selected. Only 4 studies used appropriate reference tests. In 6 studies, the reference standard was considered inadequate, and in 9 studies, it remained unclear who labeled/classified the images.

**FIGURE 2.**
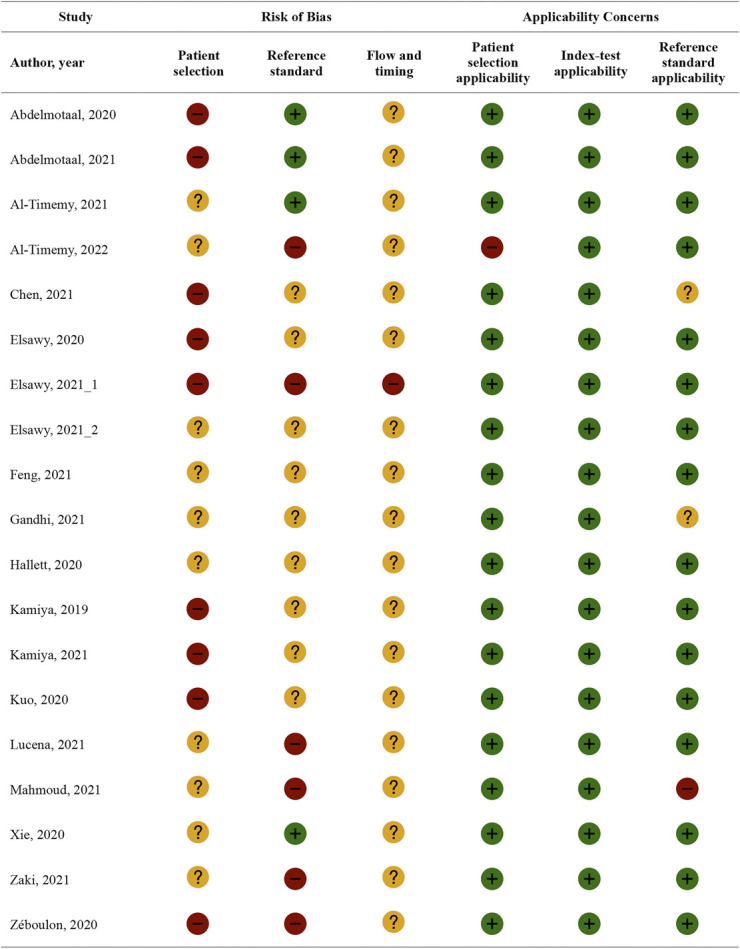
Methodological characteristics assessed by adapted QUADAS-2. + (low), − (high), and ? (unclear).

Six studies reported that imaging material or participants were excluded because of the provision of poor imaging quality and artifacts. For the remaining studies, it is unclear whether low-quality images were excluded. A summary of methodological quality assessment is shown in Figure 2 and a detailed QUADAS-2 assessment is available in Supplemental Digital Content 1 (see Table, http://links.lww.com/ICO/B627).

### DL Models and Validation Methods

The 19 included studies trained and evaluated 45 DL models with 19 different model architectures. Thirteen of 17 studies used transfer learning approaches, and in 2 studies, it was unclear whether transfer learning was used. The most common model was the adapted VGG16 model, which was evaluated by 8 studies.^12,16–18,21,23,24,27^ To train the model and evaluate the performance, 11 studies split the samples into training and validation and/or test sets. Six studies used cross-validation,^5,15,17,21,23,26^ and 2 studies used both cross-validation and split sampling technique.^7,20^ The median sample size used to evaluate model performance was 179 images/eyes (ranging from 24 to 1104 images/eyes).

### Test Performance

Of 8 studies using Scheimpflug topography images or parameters for keratoconus detection, 2 allowed calculating test performance parameters. The sensitivity ranged from 0.95 to 1.00 and specificity ranged from 0.63 to 1.00. The reported or calculated accuracy of all 8 studies ranged from 0.74 to 0.99. For studies using Placido disk-based topographies, sensitivity ranged from 0.92 to 1.00, specificity ranged from 0.91 to 1.00, and accuracy from 0.93 to 1.00. Four of 5 studies with anterior segment OCT provided sufficient information to calculate 2 × 2 tables. Sensitivity ranged from 0.94 to 0.98 and specificity ranged from 0.91 to 1.00. The accuracy of all studies using anterior segment OCT ranged from 0.85 to 0.99. The sensitivity of 2 studies reporting models using smartphone images ranged from 0.90 to 0.95 and specificity ranged from 0.95 to 0.99. Several studies subclassified keratoconus cases into different stages, but only 4 studies used the well-established Amsler-Krumeich classification. The overall accuracy ranged from 0.74 to 0.87 for studies using the Amsler-Krumeich classification. Detailed results are provided in Tables 2 and 3.

**TABLE 3.** Amsler-Krumeich Classification Performance of DL Algorithms

Author, yr	Label of Classification	Group Size	Total Sample	Sensitivity	Specificity	AUC	Accuracy
Chen et al^21^	A-K classification overall						0.774
	Healthy	32	532	0.875	0.966		0.961
	A-K classification stage 1	83	532	0.663	0.958		0.912
	A-K classification stage 2	161	532	0.789	0.868		0.844
	A-K classification stage 3	64	532	0.531	0.929		0.882
	A-K classification stage 4	192	532	0.875	0.994		0.951
Hallett et al^22^	A-K classification overall	n.r.	24				0.739
	Healthy						
	A-K classification stage 1	n.r.	24			0.917	
	A-K classification stage 2	n.r.	24			0.864	
	A-K classification stage 3	n.r.	24			0.825	
	A-K classification stage 4	n.r.	24			0.913	
Kamiya et al^5^	A-K classification overall						0.874
	Healthy	239	543	1.000	0.984		0.991
	A-K classification stage 1	108	543	0.889	0.977		0.959
	A-K classification stage 2	75	543	0.680	0.951		0.913
	A-K classification stage 3	42	543	0.714	0.952		0.934
	A-K classification stage 4	79	543	0.747	0.987		0.952
Kamiya et al^23^	A-K classification overall						0.785
	Healthy	170	349	0.988	0.944	0.997	0.966
	A-K classification stage 1	54	349	0.611	0.966	0.955	0.911
	A-K classification stage 2	52	349	0.615	0.912	0.899	0.868
	A-K classification stage 3	23	349	0.391	0.957	0.888	0.920
	A-K classification stage 4	50	349	0.640	0.950	0.943	0.905

A-K classification, Amsler-Krumeich classification; AUC, area under the curve.

## RESULTS FROM THE HIERARCHICAL SUMMARY ROC ANALYSIS

**FIGURE 3.**
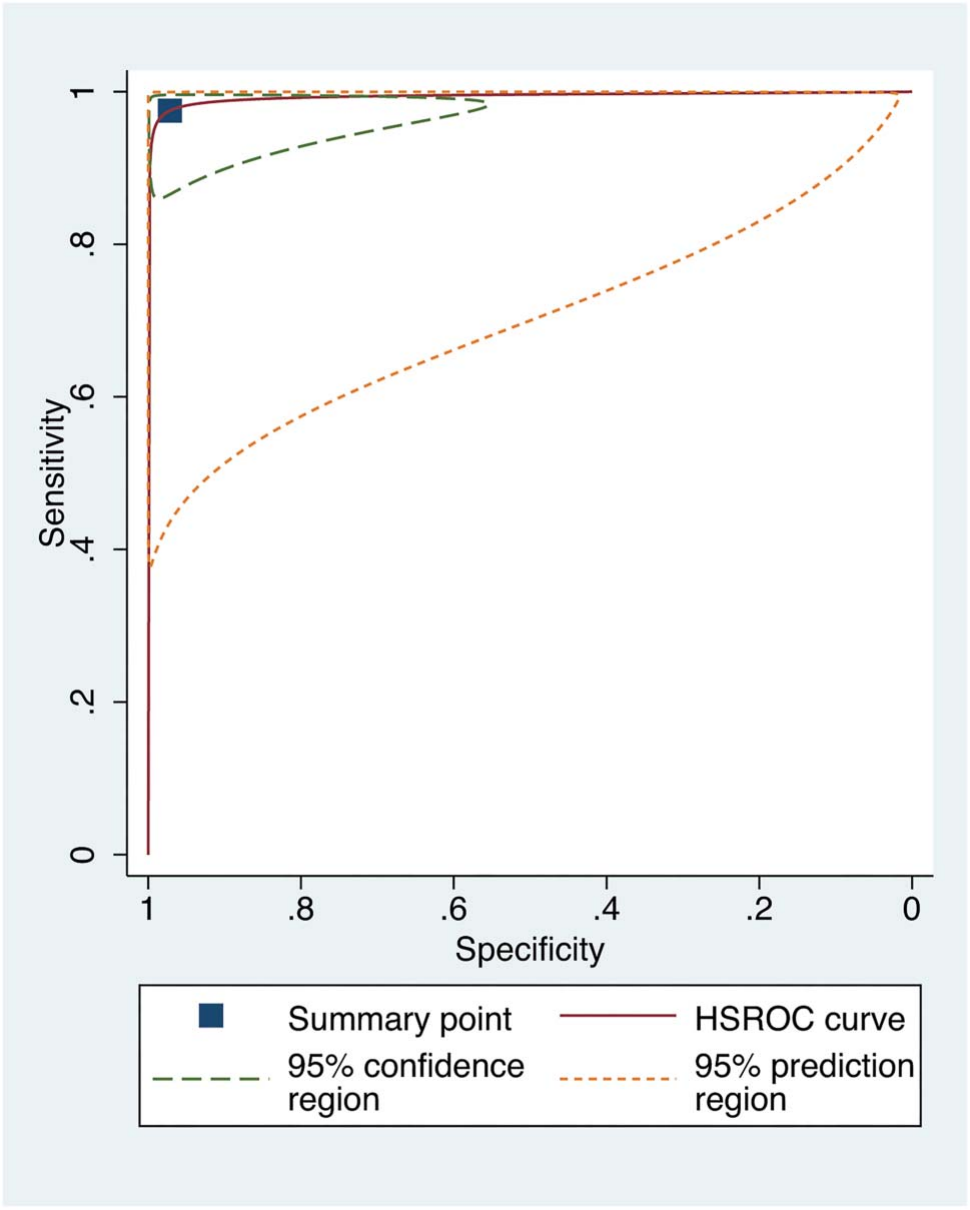
Hierarchical summary ROC curve of studies assessing models using Scheimpflug or Placido disk-based corneal topography inputs.

**FIGURE 4.**
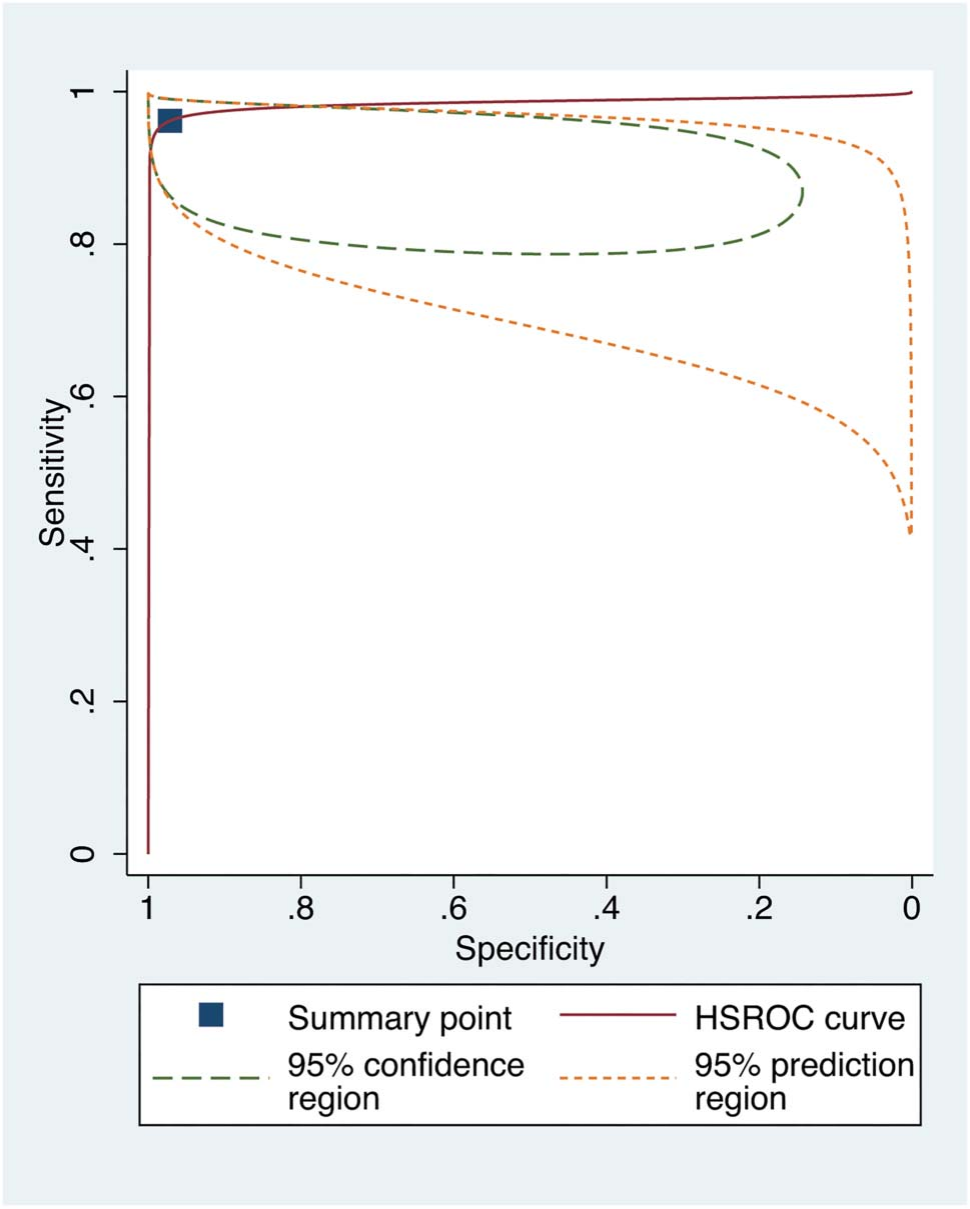
Hierarchical summary ROC curve of studies assessing models using anterior segment OCT inputs.

Of the 12 studies that allowed the extraction of contingency tables, 10 were included in the exploratory meta-analysis. Studies evaluating models with smartphone images were excluded from the quantitative analysis because of the small number of studies. We found a pooled sensitivity of 97.5% (95% confidence interval [CI], 93.6%–99.0%) and a pooled specificity of 97.2% (95% CI, 85.7%–99.5%) using Scheimpflug or Placido disk-based corneal topography inputs. For models assessing anterior segment OCT images, we found a pooled sensitivity of 96.2% (95% CI, 93.2%–97.9%) and a pooled specificity of 97.1% (95% CI, 86.3%–99.5%). Hierarchical summary ROC curves are depicted in Figures 3 and 4.

## DISCUSSION

### Main Findings

This systematic review and exploratory meta-analysis found that the overall diagnostic performance of DL models to detect keratoconus was good, but the methodological quality of included studies was modest. The lack of appropriate reference standards and reporting on patient and image selection limits the value of these DL models and hinders their translation into clinical practice.

### Results in the Light of Existing Literature

Over the past years, research on AI to detect keratoconus has grown steadily. However, to the best of our knowledge, this is the first comprehensive assessment of studies investigating the test performance of corneal imaging to detect keratoconus using a DL algorithm. This systematic review shows promising results of DL algorithms to diagnose keratoconus, but due to the poor reporting, it remains unclear how this will be translated into clinical practice.

Lin et al^29^ reviewed ML techniques in keratoconus detection and found it to be a rapidly growing field with significant potential. However, different ML methods (automated decision trees, support vector machines, and neural networks) were considered difficult to compare because keratoconus represents a broad spectrum of disease, and the lack of a large public data set prevents a comparison of newly developed models with current models and hinders the advancement of the field. In addition, Ting et al^4^ described the diagnostic performance of many AI algorithms as favorable but noted that many of them were trained with small samples and relatively few have been validated in real-world settings, which usually contain a more heterogeneous patient cohort. Furthermore, our systematic inventory shows a heterogeneous and sparse reporting and therefore the risk for bias, which brings the urgent need for adherence to reporting guidelines for AI in medical research into play.^30^ These guidelines lay out a pragmatic pathway for rigorous evaluation not only of the efficacy of an algorithm but also its effectiveness, equity, and safety.

### Strength and Limitation

This systematic review applied state-of-the-art methodology of PRISMA statement recommendations^8,9^ and assessment of study quality was performed according to adapted QUADAS-2.^10^ We relied on a comprehensive number of medical and nonmedical databases to identify eligible studies limiting the risk of selection bias.

Only an exploratory meta-analysis was feasible because of the limited availability of studies using the same input (images/parameters). In addition, only 12 of 19 studies permitted reconstructing the 2-by-2 tables because of scarce reporting. In this respect, it may be justified to repeat these analyses when additional data are available. It is plausible that reporting and publication bias also exists because algorithms with negative or unsatisfactory performance are less likely to be reported and published. Only 9 of 19 studies reported how the images were labeled; this might induce further bias as high inconsistency among experts in interpretation of ophthalmic images has been reported.^31^

### Implication for Research and Practice

Ophthalmic imaging has been at the forefront of AI advancements in clinical medicine. Application of AI technology in keratoconus management holds the potential to improve detection of the disease and monitoring of its progression. With the increasing prevalence of keratoconus, the use of AI would alleviate clinical workloads because it allows broad disease screening and detection in an objective and efficient manner. This is clinically becoming further relevant as early detection of keratoconus can improve overall visual outcome. Therefore, health care systems with minimal staff may benefit from these modern automated imaging classification algorithms to provide better patient care.

Although this inventory of studies shows promising accuracy of DL, most of the algorithms were trained and evaluated with a small sample of imaging data, which limits their clinical application as keratoconus presents in very heterogeneous phenotypes. In addition, only 4 studies used a separate test set evaluating the performance in an external validation set.^15,16,18,19^ In most of the studies, it remained unclear whether the patient sample was representative of a real-world setting. Therefore, establishing a public, high-quality, real-world data set is inevitable for developing and training highly accurate DL algorithms. A consistent data set representative of a patient population seen in clinical practice will allow for standardization and comparison of study outcomes. Furthermore, a public data set enables DL scientists worldwide to test new techniques to advance the accuracy of predictive analytics. Finally, it will be important to update the systematic review including newly published evidence^32–37^ to capture progress in model development and to monitor reporting quality and guideline adherence.

The translation of these techniques into clinical practice depends on the integration of the algorithms into popular imaging modalities, such as topography devices or anterior segment OCTs. This currently missing integration step limits the usefulness of these algorithms for clinical application but is likely to improve through licensing agreements between AI developers and device manufacturers.

## CONCLUSIONS

AI and DL algorithms hold the potential to revolutionize keratoconus diagnosis and management by performing classifications of corneal images for clinical experts by rapidly reviewing large amounts of images. Current DL algorithms show a good overall diagnostic performance, but the methodological quality of included studies is modest. There is an urgent need for a public, high-quality, real-world data set that is representative of the patient population in clinical practice to achieve major advances in the accuracy of predictive analytics.

## Supplementary Material
