# Supplementary material for: Deep Learning Models Used in the Diagnostic Workup of Keratoconus: A Systematic Review and Exploratory Meta-Analysis
Source: Cornea. 2024 Feb 1;43(7):916–31. doi: 10.1097/ICO.0000000000003467 (PMC11142647; doi:10.1097/ICO.0000000000003467)
Supplement: Supplementary file 1 [file cornea-43-916-s001.docx]

**Supplementary Table 1** Detailed methodological characteristics assessed by adapted QUADAS-2

| **Author, year** | **Consecutive/random*** | **No case-control study*** | **No inappropriate exclusions*** | **Patient selection applicability†** | **Index-test applicability†** | **Adequate reference standard*** | **Reference-test blinded*** | **Reference standard applicability†** | **All patients receive reference test*** | **All patients receive same reference test*** | **All patients included in analysis*** |
| --- | --- | --- | --- | --- | --- | --- | --- | --- | --- | --- | --- |
| Abdelmotaal, 2020 | - | ? | ? | + | + | + | + | + | + | + | ? |
| Abdelmotaal, 2021 | - | ? | ? | + | + | + | + | + | + | + | ? |
| Al-Timemy, 2021 | ?, n/a (test set) | ?, - (test set) | ? | + | + | + | + | + | + | + | ? |
| Al-Timemy, 2022 | ? | ? | ? | - | + | - | + | + | + | ? | ? |
| Chen, 2021 | n/a | - | ? | + | + | ? | + | ? | + | ? | ? |
| Elsawy, 2020 | n/a | - | ? | + | + | ? | + | + | + | ? | ? |
| Elsawy, 2021_1 | - | + | ? | + | + | - | + | + | + | - | - |
| Elsawy, 2021_2 | ? | ? | ? | + | + | ? | + | + | + | ? | ? |
| Feng, 2021 | ? | ? | ? | + | + | ? | + | + | + | ? | ? |
| Gandhi, 2021 | ? | ? | ? | + | + | ? | + | ? | + | ? | ? |
| Hallett, 2020 | ? | + | ? | + | + | ? | + | + | + | ? | ? |
| Kamiya, 2019 | n/a | - | ? | + | + | ? | + | + | + | ? | ? |
| Kamiya, 2021 | n/a | - | ? | + | + | ? | + | + | + | ? | ? |
| Kuo, 2020 | n/a | - | ? | + | + | ? | + | + | + | ? | ? |
| Lucena, 2021 | ? | ? | ? | + | + | - | + | + | + | ? | ? |
| Mahmoud, 2021 | ? | ? | ? | + | + | - | + | - | + | ? | ? |
| Xie, 2020 | ?, n/a (test set) | +, - (test set) | ? | + | + | + | + | + | + | + | ? |
| Zaki, 2021 | ? | ? | ? | + | + | - | + | + | + | + | ? |
| Zéboulon, 2020 | n/a | - | ? | + | + | - | + | + | + | + | ? |
